# Fasting Induces the Expression of PGC-1α and ERR Isoforms in the Outer Stripe of the Outer Medulla (OSOM) of the Mouse Kidney

**DOI:** 10.1371/journal.pone.0026961

**Published:** 2011-11-04

**Authors:** Christina T. Teng, Yin Li, Pat Stockton, Julie Foley

**Affiliations:** 1 Biomolecular Screening Branch, National Institute of Environmental Health Sciences, National Institutes of Health, Research Triangle Park, North Carolina, United States of America; 2 National Toxicology Program and Laboratory of Reproductive and Developmental Toxicology, National Institute of Environmental Health Sciences, National Institutes of Health, Research Triangle Park, North Carolina, United States of America; 3 Cellular and Molecular Pathology Branch, National Institute of Environmental Health Sciences, National Institutes of Health, Research Triangle Park, North Carolina, United States of America; Institut de Génomique Fonctionnelle de Lyon, France

## Abstract

**Background:**

Peroxisome proliferator-activated receptor-γ co-activator-1α (PGC-1α) is a member of the transcriptional coactivator family that plays a central role in the regulation of cellular energy metabolism under various physiological stimuli. During fasting, PGC-1α is induced in the liver and together with estrogen-related receptor a and γ (ERRαand ERRγ, orphan nuclear receptors with no known endogenous ligand, regulate sets of genes that participate in the energy balance program. We found that PGC-1α, ERRα and ERRγ was highly expressed in human kidney HK2 cells and that PGC-1α induced dynamic protein interactions on the ERRα chromatin. However, the effect of fasting on the expression of endogenous PGC-1α, ERRα and ERRγ in the kidney is not known.

**Methodology/Principal Findings:**

In this study, we demonstrated by qPCR that the expression of PGC-1α, ERRα and ERRγ was increased in the mouse kidney after fasting. By using immunohistochemistry (IHC), we showed these three proteins are co-localized in the outer stripe of the outer medulla (OSOM) of the mouse kidney. We were able to collect this region from the kidney using the Laser Capture Microdissection (LCM) technique. The qPCR data showed significant increase of PGC-1α, ERRα and ERRγ mRNA in the LCM samples after fasting for 24 hours. Furthermore, the known ERRα target genes, mitochondrial oxidative phosphorylation gene COX8H and the tricarboxylic acid (TCA) cycle gene IDH3A also showed an increase. Taken together, our data suggest that fasting activates the energy balance program in the OSOM of the kidney.

## Introduction

Nutrient balance and metabolic homeostasis in mammals are maintained by an intricate regulatory circuitry which is controlled by transcriptional mechanisms. During times of fasting, a primary fuel source shift from carbohydrate to ketone bodies occurres in the liver and kidney. This change is accompanied by increase of gluconeogenesis and fatty acid oxidation [1], [2], [3]. Energy homeostasis in the liver has been well studied however the kidney has been lesser investigated. The major functions of the kidney are monitored by autoregulation [4], [5] and the endocrine system [6], [7] in order to maintain a proper body fluid equilibrium and homeostasis of the whole body. Furthermore, kidney also plays a role in energy balance because this organ possesses sufficient gluconeogenic enzyme activities and contributes substantial amount of glucose during fasting [3]. Consistent with the enhanced gluconeogenesis the pyruvate dehydrogenase kinase 4 (PDK4), a key regulatory enzyme involved in switching the energy source from glucose to fatty acids, was upregulated in kidney while fasting [8]. The kidney also contains genes regulated by circadian rhythm whose relative expression levels are significantly affected by time of day and the feeding status. Their expression level could be further modified by fasting [9], [10].

The kidney is a complex organ, with different regions carrying out specific activities while responding differently to various physiological conditions. Because of this functional division, studies carried out at the whole-kidney level cannot define sites and mechanisms of physiological processes. To gain a molecular understanding of kidney function, the human kidney was micro dissected into the glomerulus and seven different nephron segments and their transcriptomes were characterized. [11]. Based on the data, a high-resolution map on gene expression was established and revealed a correlation between sets of genes expressed and the known function of the kidney at that region. The outer stripe of the outer medulla (OSOM), region adjacent to the renal cortex, plays a critical role in kidney function in reabsorption and pressure maintenance. This region is very sensitive to environmental insults and is the target of many renal disorders such as tissue damage caused by an increase of systemic pressure [12], [13], [14] and vitamin D-deficient-induced down regulation of type II Na^+^-dependent Pi transporter (NaPi-2) protein and mRNA [15]. This region also contains the hypoxia-inducible factor (HIF-2α) regulated erythropoietin-producing (EPO) cells [16] [17], [18], the mineralocorticoid receptors, the 11β-dehydrogenase [19] and peroxisomal enzymes [20]. It was found using global transcription profiling analysis that estradiol-17β (E2) affects the expression of a large number of genes in rodent kidney [21]. The same study showed that numbers of E2-induced genes such as the activator of transcription 5A (STAT5A), tissue factor (TF), GADD45G and cytochrome P450 family gene CYP7B1 were localized to the OSOM of the kidney by *in situ* hybridization [21]. These studies demonstrate that several hormones have influence on the function at this region.

Recently, peroxisome proliferator-activated receptor γ coactivator 1α (PGC-1α), a transcriptional coactivator, was discovered to function as a master controller of energy balance and nutrient homeostasis [22], [23]. This coactivator coordinates with transcription factors and regulates sets of genes that participate in metabolic pathways and biological processes in a tissue-specific manner. PGC-1α and its family members are highly responsive to a variety of environmental cues such as changes of temperature, nutritional status or physical activity (see review and reference therein [24]). Previously, we demonstrated that PGC-1α and ERRα and ERRγ (members of the NR3B orphan nuclear receptor subgroup) [25] are highly expressed in the kidney [26] and that PGC-1α induced the expression of the ERRα gene in HK2, human kidney cells [27]. Although PGC-1α interacts with many nuclear receptors and transcription factors [23] to regulate the energy balance program, its interaction with ERRα is unique and has important functional implications because PGC-1α has a protein surface that is dedicated to the ERRs interaction [28], [29]. Moreover, ERRα was discovered to be a key transcription factor involved in cross-talk with PGC-1α in regulating the mitochondrial biogenesis and the oxidative phosphorylation program by directly influencing the expression of gene subsets participating in these activities (see review and references therein [30], [31]). In addition to PGC-1α, ERRα was also induced in mouse liver upon fasting [26], [32]. Under long term caloric restriction, ERRα expression was found to be increased in kidney, small intestine, heart and skeletal muscle to varying degrees [33]. The presence of a high level of PGC-1α and its regulating partners, the ERRs, in the kidney suggests that these factors could play a major role in the metabolic status of this organ. In this study, we showed that a short term fasting indeed induces PGC-1α, ERRα and ERRγ expression in the kidney with the highest expression occurring in OSOM of the kidney. In addition, by using laser capture technique, we demonstrated that the expression of selected ERRα target genes involved in tricarboxylic acid (TCA) cycle and mitochondria oxidative phosphorylation were also induced.

## Materials and Methods

### Ethics Statement

All animal care and use procedures were in accordance with NIH guidelines. The animal study protocol (#88–32, LRDT) was approved by NIEHS Animal Care and Use Committee.

### Animal fasting and sample preparation

Female CD-1 mice 36-days of age (Charles River Laboratories, Wilmington, Mass.) were housed in the National Institute of Environmental Health Sciences (NIEHS) animal laboratories (at 23°C, 40–60% humidity, and 0600 to 1800 hrs light cycles) and provided with unlimited water and food (NIH31 chow). For fasting, food was removed at 6 PM and the animals were sacrificed at 6PM (24 h) the next day. The control mice (fed ad libitum) were housed in the same facility and sacrificed at 6PM together with the 24 hs fasting mice. Each experimental group consisted of three mice. The kidneys were removed and one kidney from each mouse was immediately fixed in Bouin's fixative and processed for immunohistochemistry (IHC) study and the other kidney from the same mouse was frozen on dry ice and stored at −80°C until RNA extraction.

### Transient transfection and Western blotting analysis

HeLa cells were seeded overnight in the 60-mm culture dishes. Two µg of pcDNA3-vector, pcDNA-ERRα, pcDNA-ERRγ or pcDNA-myc-ERRγ expression plasmids were transfected using the Effectene transfection reagent (QIAGEN, Valencia, CA) for 48 hrs. Whole cell lysates were prepared by using BD TransFactor Extraction Kits according to the supplier's protocol (BD Biosciences, Palo Alto, CA). The protein concentrations were determined by using Bio-Rad Protein assay reagents (Pierce, Rockford, IL). The samples were heated at 95°C for 5 min and 40 µg of proteins were loaded onto each lane of a freshly made 10% SDS-PAGE gel. The proteins were separated by electrophoresis and then electrotransferred onto polyvinylidene difluoride membranes. The membranes were blocked in PBS-T (Phosphate Buffered Saline containing 0.1% tween-20) and 5% nonfat milk for 2 hrs at room temperature and the primary antibody for ERRα N-terminal peptide (P2, GSSETETEPPVALAPGPAPTR) or C-terminal peptide (P3, SVHIEDAEAVEQLREALHEALLEYEA) [34], ERRγ N-terminal peptide (pG, LYPSAPILGGSGPVRKLYDDCSS) [27], c-Myc (9E10, Santa Cruz biotechnology, Santa Cruz, CA) or β-actin (monoclonal clone AC-74, Sigma, St. Louis, MO) was then added into the PBS-T with continuous incubation overnight at 4°C. Unbound primary antibodies were rinsed off with PBS-T. Horseradish peroxidase-conjugated secondary antibody at a dilution of 1∶5,000 in PBS-T/milk was added for another 1 hr of incubation at room temperature. Immunoreactive products were detected by the ECL system (Amersham Pharmacia, Piscataway, NJ). β-actin was used as a loading control.

### Immunohistochemistry (IHC)

Mouse kidneys were placed immediately into 10–13 ml of Bouin's solution after euthanasia. Tissue was rocked on an Adams Nutator (Clay Adams, Parsippany, NJ) in the Bouin's solution for 24–30 h before the Bouin's-solution was disposed of and 70% ethanol was added to the tissue. Tissues were processed and embedded in paraffin once the solution remained clear in the 70% (w/v) ethanol for several hours. Embedded tissue was sectioned (thickness, 7 µm) and placed onto charged-glass microscope slides for IHC staining. The slides were deparaffinized and immunostained with the respective antibodies according to the method previously described [35]. IgG fractions of anti-ERRα (P2, 6 µg/ml), anti-ERRγ (pG) or anti-PGC-1α (H-300, Santa Cruz Biotechnology) were used as the primary antibodies, and incubation was carried out overnight at 4°C. Non-immune serum or its IgG fraction was used as the negative control for the IHC staining study. The slides were immunostained according to the manufacturer's instructions with the AEC kit (Zymed Laboratory, South San Francisco, CA) and counterstained with sterile, modified hematoxylin (Sigma, St. Louis, MO). The slides were rinsed in distilled water to remove excess hematoxylin, air-dried, and mounted by GVA mounting solution (Invitrogen, Carlsbad, CA) with a coverslip.

### Laser capture micro-dissection (LCM) of paraffin-embedded samples

Under sterile conditions, both kidneys from 5 fasting and 5 non-fasting mice were removed within 5 minutes of animal euthanasia, cut in half (crosswise) and fixed in freshly made 4% paraformaldehyde for overnight fixation (with at least 20 ml of fixative in a sterile 50 ml conical tube for each sample). Samples were processed for histology, embedded in paraffin, sectioned at 8 µm thick and stained in aqueous cresyl violet acetate to locate theOSOM. The samples were immediately laser micro-dissected under sterile conditions using the Molecular Machines and Industries Cellcut® microscope (MMI, Inc., Haslett, MI.) and collected from polyethylene terephthalate (PET) foil slides (MMI, Inc., Haslett, MI) following the NIEHS Laser Microdissection Core Facility guidelines. The LCM samples were lysed using PureLink™ FFPE RNA kit (Invitrogen) and stored frozen at −80°C. The RNA isolation was performed immediately after lysing LCM samples and qPCR assay were carried out within one week of sample collection.

### RNA extraction and real-time PCR

Total RNA was extracted by using PureLink™ FFPE RNA kit (Invitrogen). First-strand cDNA synthesis was performed using superscript reverse transcriptase (Invitrogen). The mRNA levels of ERRαERRγ and PGC1α were measured using SYBR green assays (Applied Biosystems). The sequences of primers used in qPCR are listed in Table 1. Each sample was quantified against its GAPDH transcript content, and then normalized with respect to the control group. The experiments were repeated three times and results are presented as fold increase ± SEM.

**Table 1:** The qPCR Primers.

Gene (accession No.)	Primers
ERRα (NM_007953)	
Forward	5′-CGG TGT GGC ATC CTG TGA-3′
Reverse	5′-GCG TCT CCG CTT GGT GAT-3′
ERRβ (NM_0011595000)	
Forward	5′-ACGGCTGGATTCGGAGAAC -3′
Reverse	5′-TCCTGCTCAACCCCTAGTAGATTC -3′
ERRγ (NM_011935)	
Forward	5′-TCA AAG CCC TCA CCA CAC TCT-3′
Reverse	5′-GCC AGG GAC AGT GTG GAG AA-3′
PGC1α (NM_008904)	
Forward	5′-GTG TTC CCG ATC ACC ATA TTC C-3′
Reverse	5′-CGG TGT CTG TAG TGG CTT GAT TC-3′
COX8H (NM_007751)	
Forward	5′-AGG AGT GCG ACC CGA GAA TC-3′
Reverse	5′-GGC TAA GAC CCA TCC TGC TGG-3′
IDH3A (NM_029573)	
Forward	5′-AGG ACT GAT TGG AGG TCT TGG-3′
Reverse	5′-ATC ACA GCA CTA AGC AGG AGG-3′
GAPDH (NM_DQ403054)	
Forward	5′-TGC ACC ACC AAC TGC TTA G-3′
Reverse	5′-GAT GCA GGG ATG ATG TTC -3′

## Results

### Fasting induces the expression of ERRα, ERRγ and PGC-1α genes in mouse kidney

**Figure 1:**
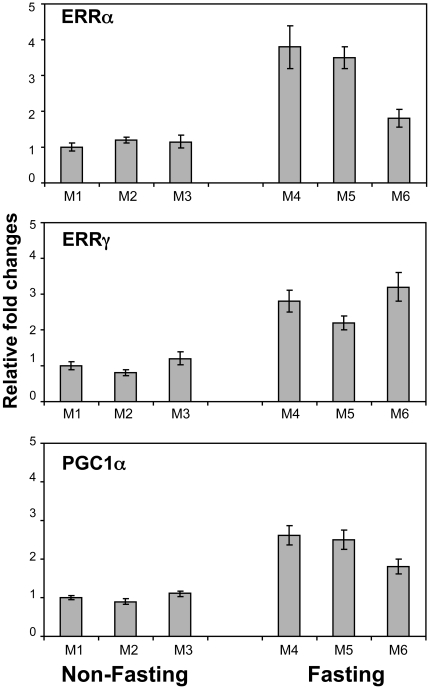
Effect of fasting on the expression of ERRα, ERRγ and PGC-1α in mouse kidney. Total RNA from nonfasting (control) and fasting mouse kidney was prepared from each individual mouse (M1-M6). Detection of ERRα, ERRγ and PGC-1α mRNA levels were conducted by qPCR as described in material and method section. Error bars represent SEM of three replicates. Average of the control sample M1 is set as 1.

**Figure 2:**
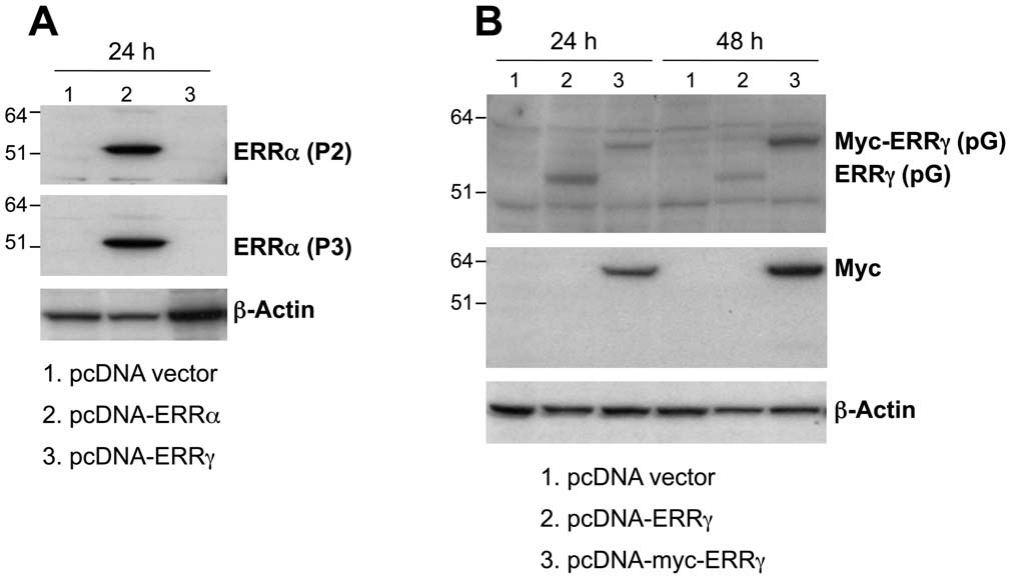
Antibody specificity. Rabbit polyclonal antibody raised against specific synthetic peptide at N-terminal (P2) and C-terminal (P3) of ERRα and ERRγ (pG), as described before was tested with Western blotting. A. P2 and P3 antibodies. B. pG and c-Myc antibodies. The cell extract was prepared from HeLa cells which was overexpressed with expression construct of ERRα and ERRγ with or without myc tag. β-actin serves as loading control.

**Figure 3:**
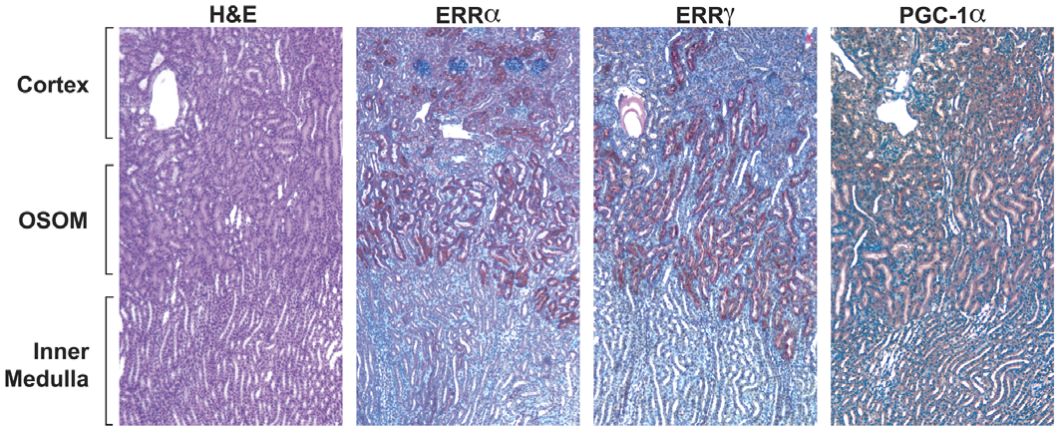
IHC staining. Paraffin-embedded control mouse kidney sections were immunostained with the P2 for ERRα, pG for ERRγ and PGC-1α antibodies.

**Figure 4:**
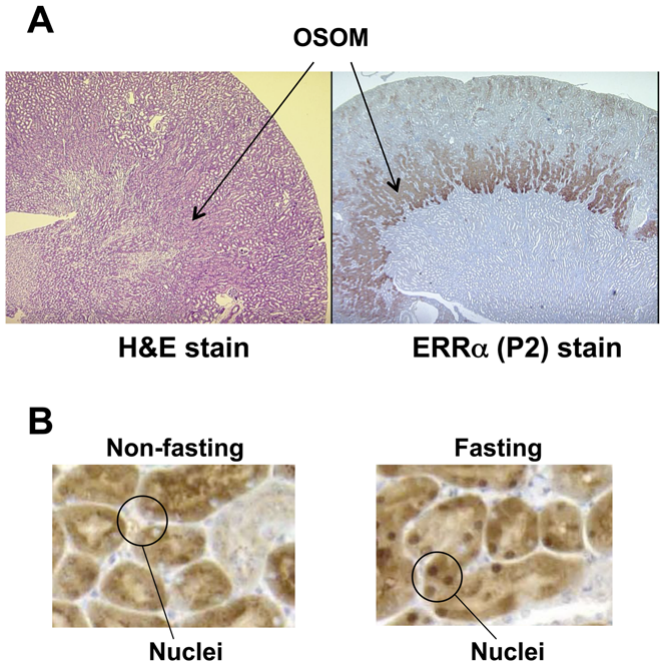
Nuclear staining of ERRα was increased in OSOM of the fasting mouse. A. The kidney sections from fasting mice were immunostained with ERRα P2 antibody (P2) or hematoxylin & eosin stained (H&E). Arrows indicate the OSOM of the fasting mice. B. High power (40x) image showed the intense staining by P2 in the nuclei of the fasting kidney as compare to the control nonfasting mouse.

In response to specific physiological cues such as cold, exercise or fasting, ERRα and PGC-1α are induced in a tissue-specific manner (review and references therein [30], [31]). Upon fasting, the demand for energy production increases, and PGC-1α and ERRα are induced in the liver to activate the downstream target genes which are involved in the oxidation phosphorylation pathway and mitochondria biogenesis [32]. We have previously reported that ERRα, ERRγ and PGC-1α are coexpressed at high levels in energy-demanding tissues such as skeletal muscle, heart and kidney [26]. To examine whether the expression of ERRα, ERRγ and PGC-1α in the kidney are also affected by short term fasting, we measured their mRNA levels using real-time PCR from both nonfasting (control) and fasting mice. As shown in Figure 1, after food was removed for 24 hr, the ERRα, ERRγ and PGC-1α mRNA increased several fold compared to the averaged control (set as 1) although variations existed among the individual animals. To examine the expression patterns of the ERRs and the coactivator PGC-1α at the cellular level, we performed IHC on the control and fasting kidney sections. The antibody specificity of ERRα and ERRγ [27], [34] is demonstrated by Western blotting (Fig. 2). Peptide derived from either human ERRα N-terminal (P2,) or C-terminal (P3) recognized the overexpressed full-length ERRα protein (Fig. 2A, lane 2) in HeLa cells but not ERRγ (Fig. 2A, lane 3). Similarly, overexpressed ERRγ with or without myc tag for 24 h and 48 h positively interacted with the antibody raised against human ERRγ N-terminal peptide (pG) (Fig. 2B, lanes 2 and 3). The P2, pG and the commercial PGC-1α antibody were used in the IHC study (Fig. 3 and 4). We found intense immunostaining for ERRα and ERRγ at the OSOM while the PGC-1α staining was less localized and also extended into other regions of the kidney. While examining the kidney sections from the fasting animals, we noticed that the immunostaining of ERRα in the nuclei of proximal convoluted tubule (PCT) in the OSOM was significantly enhanced (Fig. 4). This observation was not found by the immunostaining of ERRγ and PGC-1α (data not shown). IHC study supports the qPCR data indicating ERRα expression in the kidney is stimulated by fasting.

### Fasting induces the expression of ERRα, ERRγ and PGC-1α genes at the OSOM of the kidney

**Figure 5:**
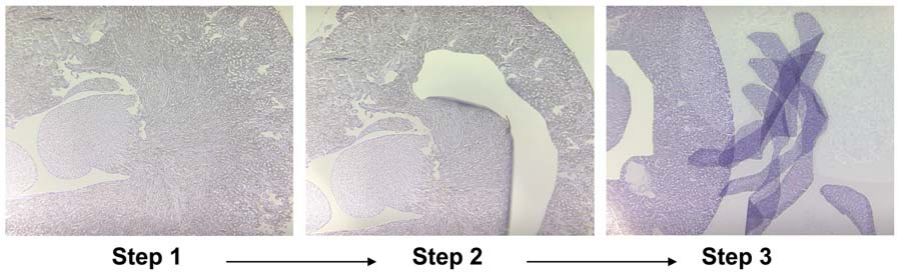
Image of laser captured samples. The OSOM was laser microdissected from the kidney sample section as described in material and method section. These samples were pooled and RNA extracted. Step 1 shows the whole kidney section. Step two shows the microdissected region. Step 3 shows the samples collected from the microdissections with remaining tissue in the background.

**Figure 6:**
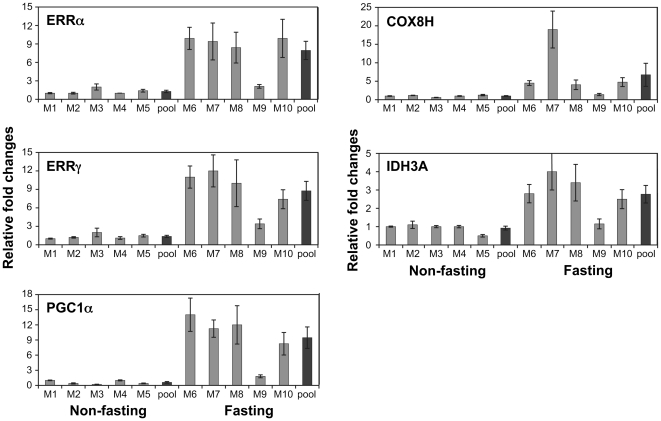
Fasting induces ERRα, ERRγ, PGC-1α, and ERRα downstream target genes (COX8H and IDH3A) expression in OSOM of the mouse kidney. Error bars represent SEM of three replicates. Average of the control sample M1 is set as 1.

The immunostaining study showed that fasting has a major effect on the OSOM of the kidney. To demonstrate this effect at the molecular level, we used LCM technique to target collection of the OSOM. RNA was isolated from the LCM sample of both nonfasting and fasting animals and analyzed by qPCR (Fig. 5). We re-examined the expression levels of ERRα, ERRγ and PGC-1α genes in the LCM samples by qPCR. Four of the 5 fasting LCM kidney samples (M6, M7, M8 and M10) showed a 9 to 12-fold increase in expression of these genes (Fig. 6 left panel) as compared to the LCM nonfasting samples (M1- M5). Fasting also induces the expression of the well known PGC-1α and ERRα target genes that are involved in various metabolic pathways such as those involved in mitochondrial oxidative phosphorylation (COX8H) and the TCA cycle (IDH3A). Although fasting induces a different response from individual animals, the pooled samples demonstrated 10-fold increase of PGC-1α and ERRs while the other genes increased 3–5-fold. These results support the earlier study on whole kidney that demonstrated fasting has an impact on the kidney metabolism and function. Additionally, the activation of PGC-1α and ERRα and γ induced by fasting primarily occurred at the OSOM. We also examined ERRβ expression in the LCM samples and found moderate increase of 2 to 3 fold (data not shown). We do not have immunostaining data on the distribution of ERRβ in mouse kidney therefore it is not known whether the increase of ERRβ mRNA level is reflecting the overall increase in the kidney or primarily at the OSOM.

## Discussion

The current investigation has several novel observations. First, fasting induces the expression of coactivator PGC-1α and orphan nuclear receptor ERRα and ERRγ in mouse kidney. Second, the fasting-induced expression of the PGC-1α, ERRα and ERRγ is primarily localized in the OSOM of the kidney. Third, genes involved in energy balance are upregulated at the OSOM of the kidney during short-term fasting.

It is well known tissues requiring a high level of energy for normal physiological function also contain greater levels of PGC-1α, ERRα and ERRγ (see review and references therein [30], [31], [36], [37]). We have examined the relative levels of PGC-1α and the ERRs in the same polyA RNA preparation of various human tissues by Northern blot analysis and found that the kidney and heart contain relatively high levels of all three mRNAs. This underscores the importance of PGC-1α, ERRα and ERRγ in maintaining the normal physiological functions in these high energy demanding tissues [26]. Interestingly, these proteins are relatively low in the liver but highly inducible during fasting [26], [32]. In this study, we found that the expression characteristics of PGC-1α, ERRα and ERRγ in the mouse kidney are reminiscent of that in the liver with an increase in expression during fasting (Fig. 1) despite already high basal levels. By using the ERRα knockout mouse model, the authors have demonstrated that several genes involved in blood pressure regulation, ion transport and electrolyte homeostasis was dysregulated. To further evaluate the participation of ERRα with the genes of the kidney that regulate the renin-angiotensin-aldosterone system, the siRNA knockdown of ERRα in the immortalized juxtaglomerular As4.1 cell line was performed and a direct repressive effect of ERRα on renin expression was confirmed [36]. These findings suggested that the ERRs are not only involved in general energy production but also involved in specific kidney functions. When we examined by qPCR, the mRNAs prepared from the OSOM of the kidney during fasting for additional target genes, we found that the expression of NHE-3 was significantly increased (Data not shown). NHE-3 plays an important role in the homeostasis of Na^+^-fluid volume. The NHE-3 Na^+^/H^+^ exchanger deficiency mice showed sharp reduction in HCO_3_^−^ and fluid absorption in the proximal convoluted tubule [38]. This observation is consistent with the role of proximal tubule in the ATP-dependent re-absorption function of the kidney. Whether ERRs play any role in NHE-3 expression is unclear and further experimentations are needed. However, we scanned the genomic sequence of mouse Slc9a3 (NM_001081060) plus its 20-kilobase pair upstream promoter sequence (65,841 bp in total) and found several putative ERE/ERREs. Although it is not clear if any of the predicted response elements functional and could bind ERRs, the possibility exists that NHE-3 gene could be regulated by PGC-1α/ERRs pathway.

Study on ERRγ null mice revealed the gene expression of key potassium channel subunits in the embryonic kidney were markedly reduced and the potassium homeostasis was profoundly dysregulated [39]. The study highlights the important role that ERRγ plays in the control of ion homeostasis in highly oxidative renal tissue. It is not surprising that both ERRα and ERRγ carry out similar physiological roles in the kidney since they share 98% identity in their DNA binding domain, are able to form homodimers or heterodimers with each other, and bind to their target genes in a similar fashion [26], [40] implicating that these two receptors could target the same gene and carry out comparable cellular energy metabolism functions. These observations reconfirm the functional roles of ERRs when coexpressed in the kidney under normal and stressful physiological conditions.

Inducing PGC-1α expression in the liver and muscle by fasting has been well documented [1], [32], [37], [41], [42], [43] and our current study is able to add the kidney to this list. PGC-1α is a key regulator to metabolic homeostasis and it activates glycogenolysis, switches on gluconeogenesis and increases fatty acid oxidation by activating the transcriptional program of the genes involved in such programs during fasting. The functional mechanisms of PGC-1α rely on its ability to coactivate its DNA binding partners, the ERRs [31], [36] to transactivate a number of key enzymes involved in various metabolic functions. For instance, enzymes involved in the initial steps of steroidogenesis [41], enzymes that commit cholesterol to the neutral bile acid biosynthesis pathway [44] and enzymes that switch the energy source from glucose to fatty acids [45]. Induction of PGC-1α and its' ERR partners in the kidney during fasting strongly suggests that the energy homeostasis program in the kidney is carried out in a similar fashion as with the other tissues.

What affects the function of ERRs could also have an impact on the response and function of the kidney during fasting. Recently, it has been demonstrated that endocrine disruptors such as bisphenol A [46], [47] binds and opposes 4-OHT induced effects on ERRγ. Therefore, the effect of environmental pollutants on the function of ERRs during fasting could be an important area for further study. It has been reported that there are gender differences in lipid kinetics during the short-term fasting [48]. The basal lipolytic rate in females is higher than in males. During the short term fasting, the relative increase in whole body lipolytic rate was blunted in females as compared with males whereas the decline in glucose production was similar in both genders. There are apparent differences in the metabolic profiles of males and females in response to fasting [49], [50]. Although gender differences could contribute to the levels of gonadal hormones or body fat content, the basic mechanism of gene expression in response to fasting may differ between males and females. Our current study was carried out with female mice. Future studies will be conducted to investigate the effects of fasting on the expression of PGC-1α and the ERRs in the kidney of the male mice.
